# Impulsivity and aggression in suicide across age and sex: case–control study

**DOI:** 10.1192/bjo.2025.10076

**Published:** 2025-08-01

**Authors:** Sergio Sanz-Gómez, Constanza Vera-Varela, Diego de-la-Vega-Sánchez, María Luisa Barrigón, Adrián Alacreu-Crespo, Julio A. Guija, Ana Sánchez, Santiago de León, Enrique Baca-García, Lucas Giner

**Affiliations:** Departmento de Psiquiatría, Facultad de Medicina, Universidad de Sevilla, Seville, Spain; Department of Psychiatry, University Hospital Infanta Sofia, Madrid, Spain; Department of Psychiatry, University Hospital Virgen Macarena, Seville, Spain; Institute of Psychiatry and Mental Health, IiGSM, Hospital General Universitario Gregorio Marañón, Madrid, Spain; Departamento de Psicología y Sociología, Universidad de Zaragoza, Zaragoza, Spain; Forensic Pathology Service of the Institute of Legal Medicine and Forensic Sciences of Seville, Seville, Spain; Faculty of Information Technology, Brno University of Technology, Brno, Czech Republic; Kempelen Institute of Intelligent Technologies, Bratislava, Slovakia; Department of Psychiatry, University Hospital Jimenez Diaz Foundation, Madrid, Spain; Department of Psychiatry, University Hospital Rey Juan Carlos, Mostoles, Spain; Department of Psychiatry, General Hospital of Villalba, Madrid, Spain; Department of Psychiatry, University Hospital Infanta Elena, Valdemoro, Spain; Department of Psychiatry, Madrid Autonomous University, Madrid, Spain; CIBERSAM (Centro de Investigacion en Salud Mental), Carlos III Institute of Health, Madrid, Spain; Department of Emergency Psychiatry and Post Acute Care, CHRU Montpellier, Montpellier, France

**Keywords:** Suicide, psychological autopsy, impulsivity, aggression, sex

## Abstract

**Background:**

Impulsivity and aggression are known risk factors for suicide, with observed age and sex differences in their impact.

**Aims:**

To explore variations in impulsivity and aggression based on sex and age and examine their roles in predicting suicide.

**Method:**

We examined 582 participants (406 individuals who died by suicide, 176 non-suicidal sudden-death controls) using the psychological autopsy method. Measures of impulsivity and aggression included the Barratt Impulsiveness Scale (BIS) and the Brown–Goodwin History of Aggression (BGHA). Participants were categorised into four groups: suicide male, control male, suicide female and control female. For group comparisons, we used analyses of variance and Spearman’s rank correlation to assess the relationship between age and BIS and/or BGHA ratings. Stepwise logistic regression was used to identify predictors of suicide for each sex.

**Results:**

Higher levels of BIS and BGHA ratings were found in the suicide group compared with controls (BIS: 51.3 *v*. 42.2, *P* = 0.002, *η*^2^ = 0.017; BGHA: 7.1 *v*. 4.1, *P* < 0.001, *η*^2^ = 0.028), with no significant sex differences. BIS and BGHA ratings decreased with age in the suicide groups (suicide male: impulsivity *ρ* = −0.327, *P* < 0.001; suicide female: aggression *ρ* = −0.175, *P* = 0.038) but not among controls. Logistic regression analysis revealed that for men, aggression (odds ratio 1.072, 95% CI: 1.032–1.112) was a key predictor. For women, younger age (odds ratio 0.970, 95% CI: 0.948–0.993), low BIS impulsivity ratings (odds ratio 1.018, 95% CI: 1.001–1.036) and living with children (odds ratio 0.448, 95% CI: 0.208–0.966) were protective factors.

**Conclusions:**

Impulsive and aggressive behaviours are critical factors in suicide risk among younger individuals, indicating an age effect but no sex dimorphism, with aggressive behaviours being a better predictor for men and impulsive and aggressive behaviours for women.

Suicide is a significant global public health issue. The World Health Organization estimates that 700,000 suicides occur annually, making it the second leading cause of death for people aged 15 to 29 years.^1^ Key risk factors include prior suicide attempts and psychiatric illnesses,^2^ particularly depression, along with alcohol-related disorders, personality disorders, bipolar disorder and schizophrenia.^3^ Additional risk factors include family history of suicide, experiences of sexual or physical abuse, somatic illnesses, adverse life events and challenging social circumstances.^4^

Certain age- and sex-based differences have been identified among suicide risk factors.^5^ In Western countries, for instance, male suicide rates are three times higher than female rates,^6^ whereas the highest female suicide rates are found in Asian countries such as Bangladesh, China and Myanmar.^1^ Research on sex differences indicates that affective disorders are more prominent risk factors for female suicide, whereas substance misuse, personality disorders and childhood disorders are more significant factors among males.^7^

Suicide risk factors also vary across life stages. In adolescents, risk factors include insomnia, feelings of burdensomeness, recent conflicts with family or romantic partners, and higher prevalence of affective and personality disorder comorbidities.^8^ For adults, key risk factors are male sex, substance use comorbidities, and loss of a spouse or job.^9^ Hopelessness is a persistent risk factor for both adults and the elderly, with social factors and declining health becoming more critical with age.^10,11^

## Impulsivity and aggression in suicide

Impulsivity is strongly associated with suicidal ideation, suicide attempts and deaths by suicide.^12^ It can be conceptualised both as a state and as a trait, with impulsivity as a trait defined as the inability to resist impulses, resulting in sudden, explosive actions.^13^ Aggression, meanwhile, is defined as any motor behaviour intended to harm or injure another object or person.^14^ In the general population, impulsivity tends to be higher among males and younger individuals and is associated with various psychiatric disorders.^15^ Studies indicate that individuals who die by suicide often exhibit elevated levels of impulsivity and aggression, marking these traits as strong suicide risk factors.^16^ In addition, relatives of individuals who have died by suicide show heightened impulsive–aggressive traits, which may contribute to familial patterns of suicide.^17^

Impulsivity and aggression have key roles in suicide risk models.^18^ For instance, cluster B personality disorders, which are often marked by impulsivity, are associated with higher rates of repeated suicide attempts,^19^ whereas the lethality of attempts is linked to aggression and male sex.^20^ However, few studies have examined these factors in deaths by suicide rather than attempts, and even fewer have analysed the influence of age and sex in this context. Previous studies suggest that male individuals, particularly younger ones, who die by suicide are more likely to have exhibited impulsive and aggressive behaviour, regardless of psychiatric comorbidity.^21,22^ Research on impulsivity and aggression in suicide often focuses on a single age group. McGirr et al^23^ were the first to investigate the relationships among impulsivity, aggression and age in a representative suicide sample and found a stronger association in younger populations that diminished with age.

Using psychological autopsy, we aimed to compare levels of impulsivity and aggression by age and sex in individuals who had died by suicide versus non-suicidal sudden deaths in a sample of Caucasian participants from southern Europe. Our hypotheses were that: (a) Barratt Impulsiveness Scale (BIS) and Brown–Goodwin History of Aggression (BGHA) ratings would be higher in the suicide group than in the sudden death group; (b) BIS and BGHA ratings would be higher in males than in females; (c) the impact of impulsive and aggressive behaviours on suicide risk would decrease with age; and (d) predictors of suicide would vary by sex, with BGHA ratings having a stronger influence in men and BIS ratings in women.

## Method

### Design

The sample comprised 594 adults over 18 years of age, including 412 individuals who died by suicide and 182 non-suicidal sudden death controls (77.0% natural and 22.0% accidental deaths) from Seville, Spain (total 2022 population: Spain, 47 615 034; Seville, 1 948 393).^24^ This study is part of an ongoing case–control psychological autopsy project initiated in 2006, in which family members of all individuals who had died by suicide were invited to participate. Data for all participants were obtained from the Institute of Legal Medicine of Seville. The selection of controls followed the same methodology as for suicide cases. All eligible families were invited to participate, ensuring a comparable recruitment process across both groups. Deaths in prison or under police custody were excluded owing to limited access to detailed information about the individuals’ lives in the months or years before death. The final number of controls included was determined by the willingness of families to participate, as some declined involvement. All participants were recruited between 2006 and 2013. This time frame was chosen owing to internal changes within the Institute of Legal Medicine that occurred afterward, which restricted access to family members for participation in this study. As a result, we were unable to recruit beyond this period while ensuring that the data collection was conducted under consistent conditions.

**Fig. 1:**
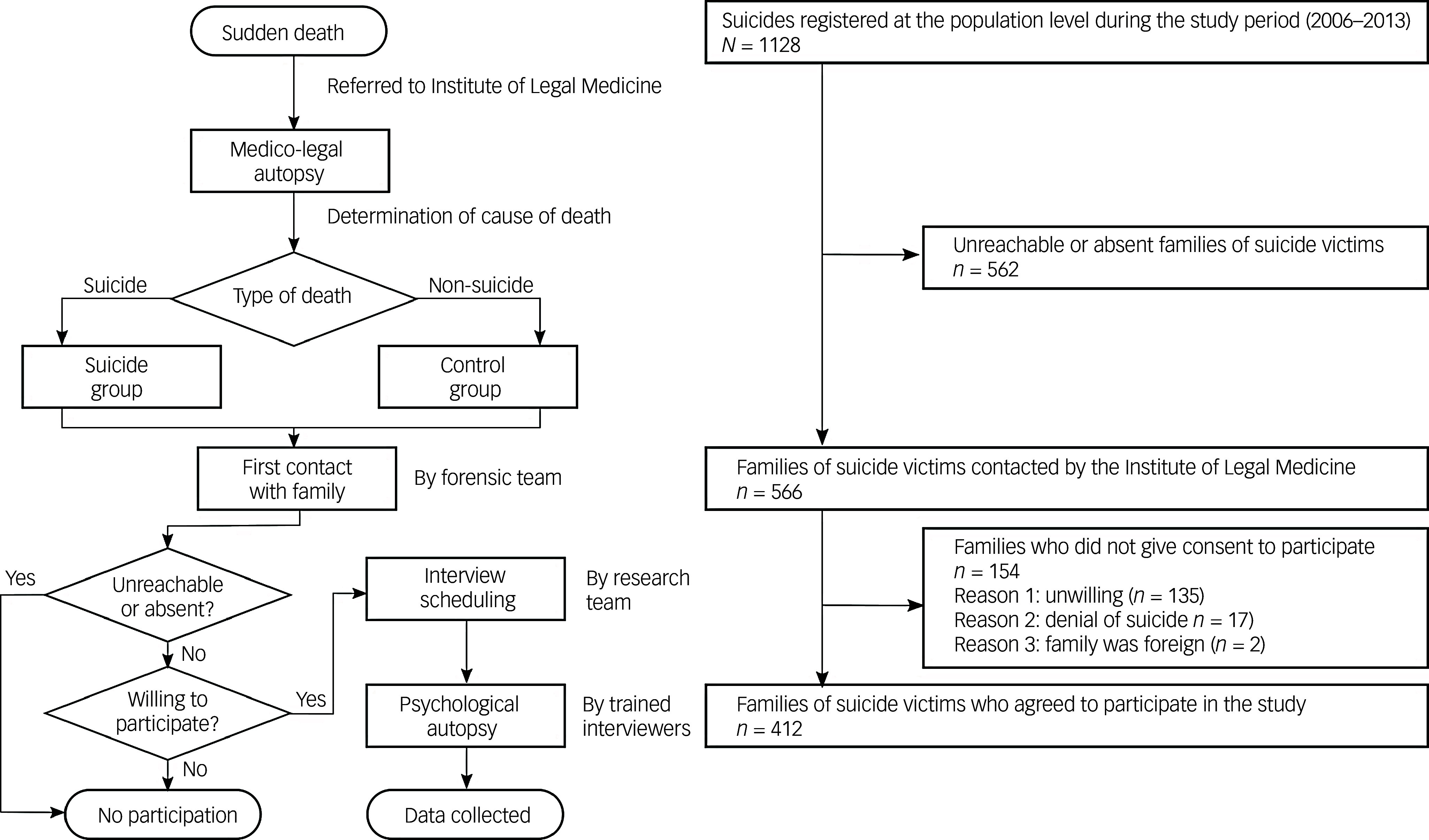
Sample selection flowchart.

Classification of deaths as suicides or non-suicides was performed by a judge and forensic physician, on the basis of findings from a forensic investigation and mandatory legal autopsy. Each death was examined using psychological autopsy, a method recommended in studies of suicide to gather information through structured interviews with family, friends and others close to the deceased.^25^ The forensic team made the first contact with family regarding the psychological autopsy protocols. If family members were willing to participate, the research team would make a second contact at least 3 months after death to arrange a meeting to carry out the psychological autopsy (Fig. 1). Interviews were conducted between 3 to 18 months after the participant’s death, primarily in-person at the University of Seville’s Department of Psychiatry, although in exceptional cases, they were held at the interviewee’s preferred location (e.g. home or workplace).

The participation rate was high among contacted family members (72.8%). However, it is important to note that in a substantial proportion of cases, family members could not be reached for an initial contact or no contact was available at all. Suicide cases registered for this study accounted for one-third of total suicides in the area during the study period, as shown in Supplementary Table 1 available at https://doi.org/10.1192/bjo.2025.10076.

All willing participants were invited to the interview, with up to seven family members interviewed per case. Although one or two relatives typically took the lead in providing information, all voices were heard, and discrepancies were clarified within the family group. In suicide cases, the main interviewee was most often a first-degree relative (sibling, parent or child; 70.6%), followed by partners (16.5%) and other relatives or close friends (12.9%). For controls, first-degree relatives made up 56.9% of interviewees, whereas partners and other relatives or friends accounted for 28.7% and 14.4%, respectively.

The psychological autopsy protocol comprised three open-ended questions about the life and personality of the deceased individual, an extensive sociodemographic and psychosocial data questionnaire, application of several standardised instruments and an assessment of the individual’s history of suicidal behaviour and of the weeks preceding death. The modules of the protocol that are relevant to this study are described in the ‘Assessment’ section. The mean duration of the interview was 2 h 25 min for suicide cases and 2 h for controls. Two psychiatrists and two psychologists, trained by the principal investigator, conducted the interviews. Each interview was followed by an interdisciplinary consensus meeting to establish a diagnosis.

The authors confirm that all procedures comply with the ethical standards of relevant national and institutional committees on human experimentation and the Helsinki Declaration of 1975, as revised in 2013. The University of Seville’s Ethics Committee (institutional review board) approved all procedures involving human participants (approval number 5012008), and all interviewees provided written informed consent before participating.

### Assessment

Collected sociodemographic variables included sex, age, partnership status (yes/no), living with children (yes/no), living alone (yes/no) and employment status. Psychiatric diagnoses were determined using the Spanish versions of the SCID-I and SCID-II structured interviews, based on DSM-IV criteria^26,27^ Both have been validated for use in psychological autopsy studies.^28^

Impulsivity was assessed with the Spanish version of the BIS-11,^13,29^ consisting of 30 items across three subscales: cognitive, motor and non-planning impulsiveness. Each item is scored from 0 to 4, with higher total scores indicating greater impulsivity. No specific cut-off exists, but a median total score of 32.5 has been proposed in a Spanish sample of psychiatric patients.^29^

Aggression was measured using the BGHA,^30^ a 33-item scale evaluating aggression in the last 6 months of life and across three life stages: childhood (≤12 years), adolescence (13–18 years), and adulthood (≥19 years). For this study, only adulthood was assessed. This scale has been previously applied in psychological autopsy research.^31^ Higher scores reflect greater aggression. Validity studies have confirmed that impulsivity and aggression evaluations from informants are comparable with direct evaluation of the individual.^32^

### Control of bias

To reduce bias in our psychological autopsy protocol, several strategies were implemented. Both the suicide and control groups consisted of deceased individuals, and sampling was conducted through the same institution, ensuring consistency between groups. Interviews were conducted at least 3 months after the death, allowing informants time to reflect on their loss and reducing the impact of recall bias. Interviews were also scheduled to avoid significant dates (e.g. birthdays or holidays) that could trigger strong emotional responses and distort recall. Widely validated instruments were used to assess impulsivity and aggression, ensuring a consistent approach across cases. All researchers underwent rigorous training in the protocol and received supervision. Periodic consensus meetings were held to review individual protocols and ensure consistent interpretation.

### Statistical analysis

A descriptive analysis was conducted on demographic (sex, age, employment), psychosocial (partnership status, number of children, living alone) and personality (impulsivity and aggression) variables. Continuous variables were summarised as the mean and standard deviation and categorical variables as frequencies and percentages. The Kolmogorov–Smirnov test was used to assess normality; variables not meeting the normality assumption were log_10_ transformed. Group comparisons (suicide versus control) were performed with chi-squared tests for categorical variables and Student’s *t*-tests for continuous variables.

To explore the main effects of sex and age by group (suicide versus control) on impulsivity (BIS) and aggression (BGHA) and their interaction, separate analyses of variance were conducted for each factor (one with sex, group and sex × group; the other with age, group and age × group). Age was categorised into three groups: young adults (≤34 years), older adults (35–64 years) and elderly (≥65 years). We used Bonferroni correction for *post hoc* comparisons, and effect sizes were calculated using *η*^2^ (eta-squared) to complement *P*-values. In addition, we used Spearman’s rank correlation to assess the relationship between age and impulsivity (BIS) and aggression (BGHA) scores by sex and type of death for non-normally distributed data. Correlation coefficients (*ρ*) with statistical significance were reported, and Fisher’s correction was used to examine the relative strengths of correlations.

Separate logistic regression models were performed for males and females to assess the impact of impulsivity, aggression (independent variables: BIS, BGHA and BIS × BGHA) and sociodemographic factors (age, having a partner, living with children, living alone and employment status) on suicide group membership (suicide versus control). We used a stepwise forward selection method to identify the most significant predictors for each sex, retaining only variables with meaningful contributions to suicide risk (entry criterion *P* < 0.05), thereby reducing overfitting and enhancing interpretability. Odds ratios with 95% confidence intervals are reported, and model fit was evaluated using the Hosmer–Lemeshow test and the area under the receiver operating characteristic curve (AUC). Multicollinearity was checked with the variance inflation factor to confirm the independence of impulsivity and aggression as predictors.

Missing values were scarce (13 for the BIS and four for the BGHA, with no further missing values). Visual inspection of the data suggested that these were missing completely at random. Cases with missing values were removed before the analysis was conducted; this did not result in any significant change in study power. All analyses were conducted using SPSS (version 29.0.1.0 for Windows).

### Sensitivity analysis

A sensitivity power analysis using G*Power (version 3.1.9.4 for Windows; Heinrich-Heine University, Düsseldorf, Germany; https://www.psychologie.hhu.de/arbeitsgruppen/allgemeine-psychologie-und-arbeitspsychologie/gpower) was used to assess the minimum detectable effect size for the study. The analysis was carried out in the sample with a full data-set (406 suicide cases and 176 controls) and was based on a two-tailed independent-samples *t*-test with an *α* of 0.05 and a power (1−*β*) of 0.80. It revealed that the study could detect an effect size of Cohen’s *d* = 0.248 or larger. The equivalent *η*^2^ was 0.0152, indicating that the study had adequate power to detect an effect explaining at least 1.52% of the variance. Although the study was sufficiently powered for medium to large effects, smaller effects may have gone undetected.

## Results

The final sample consisted of 406 suicide cases and 176 controls, divided as follows: 305 male individuals who died by suicide (suicide male; 75.1% of suicides), 123 male controls (control male; 57.4% of controls), 101 female individuals who died by suicide (suicide female; 24.9% of suicides) and 53 female controls (control female; 30.1% of controls). Overall, individuals in the suicide group were younger than those in the control group (54.3 *v*. 57.9, *t* = −2.506, *P* = 0.012; Table 1).

**Table 1:** Sociodemographic and clinical characteristics of the sample by group and sex

Characteristic		Suicide group *n* = 406	Control group *n* = 176	Statistical test (suicide group versus control group)	Suicide male *n* = 305 (52.3%)	Control male *n* = 123 (21.1%)	Suicide female *n* = 101 (17.3%)	Control female *n* = 53 (9.1%)	Statistical test (four groups)
Age, years (mean)		54.33 (52.47–56.18)	57.89 (55.22–60.56)	*T* = −2.506 *P* = 0.012	53.66 (51.42–55.91)	55.45 (52.28–58.63)	56.32 (53.15–58.63)	63.55 (58.80–68.30)	*F* = 4.342 *P* = 0.005
Partner, *n* (%)	Yes	195 (48.0)	87 (49.43)	*χ*^2^ = 1.035 *P* = 0.596	149 (48.85)	61 (49.59)	46 (45.54)	26 (49.06)	*χ*^2^ = 0.433 *P* = 0.933
Children, *n* (mean)		1.93 (1.75–2.10)	2.12 (1.86–2.38)	*T* = −1.192 *P* = 0.234	1.82 (1.62–2.02)	2.05 (1.74-2.36)	2.25 (1.86-2.63)	2.286 (1.782–2.795)	*F* = 2.141 *P* = 0.094
Live alone, *n* (%)	Yes	74 (18.73)	29 (16.96)	*χ*^2^ = 0.475 *P* = 0.788	57 (19.26)	22 (18.64)	17 (17.17)	7 (13.21)	*χ*^2^ = 1.195 *P* = 0.754
Working life, *n* (%)	Active	51 (14.53)	36 (24.32)	*χ*^2^ = 7.545 *P* = 0.110	47 (18.01)	29 (28.71)	4 (4.44)	7 (14.89)	*χ*^2^ = 133.601 *P* < 0.001
	Inactive (with disability)	229 (65.24)	85 (57.43)		190 (72.79)	69 (68.32)	39 (43.33)	16 (34.04)	
	Inactive (without disability)	71 (20.23)	27 (18.24)		27 (9.19)	5 (2.97)	47 (52.22)	24 (51.06)	
Axis I lifetime, *n* (%)		354 (87.2)	109 (61.9)	*χ*^2^ = 48.481 *P* < 0.001	257 (84.3)	75 (61.0)	97 (96)	34 (64.2)	*χ*^2^ = 54.862 *P* < 0.001
	Depressive disorder	274 (67.5)	51 (29.0)	*χ*^2^ = 74.658 *P* < 0.001	189 (62.0)	33 (26.8)	85 (84.2)	18 (34.0)	*χ*^2^ = 89.759 *P* < 0.001
	Bipolar disorder	20 (4.9)	5 (2.8)	*χ*^2^ = 1.346 *P* = 0.510	11 (3.6)	3 (2.4)	9 (8.9)	2 (3.8)	*χ*^2^ = 6.652 *P* = 0.0.84
	Psychotic disorder	43 (10.6)	9 (5.1)	*χ*^2^ = 14.474 *P* < 0.001	33 (10.8)	7 (5.7)	10 (9.9)	2 (3.8)	*χ*^2^ = 4.773 *P* = 0.189
	Anxiety disorder	94 (23.2)	35 (19.9)	*χ*^2^ = 1.045 *P* = 0.593	62 (20.3)	14 (11.4)	32 (31.7)	21 (39.6)	χ^2^ = 23.553 *P* < 0.001
	Substance use disorder	141 (34.7)	44 (25.0)	*χ*^2^ = 5.830 *P* = 0.054	124 (40.7)	40 (32.5)	17 (16.8)	4 (7.5)	*χ*^2^ = 38.875 *P* < 0.001
	Eating disorder	5 (1.2)	0 (0)	*χ*^2^ = 2.199 *P* = 0.333	1 (0.3)	0 (0)	4 (4.0)	0 (0)	*χ*^2^ = 13.941 *P* = 0.003
	Others	22 (5.4)	4 (2.3)	*χ*^2^ = 2.899 *P* = 0.235	22 (7.2)	3 (2.4)	0 (0)	1 (1.9)	*χ*^2^ = 12.124 *P* = 0.007
Axis II lifetime, *n* (%)		237 (58.7)	58 (33.1)	*χ*^2^ = 32.786 *P* < 0.001	181 (40.3)	44 (35.8)	56 (55.4)	14 (26.9)	*χ*^2^ = 33.526 *P* < 0.001
	Cluster A	131 (32.3)	33 (18.8)	*χ*^2^ = 11.486 *P* = 0.003	92 (30.2)	22 (17.9)	39 (38.6)	11 (20.8)	*χ*^2^ = 13.910 *P* = 0.003
	Cluster B	144 (35.5)	22 (12.5)	*χ*^2^ = 32.201 *P* < 0.001	110 (36.1)	16 (13.0)	34 (33.7)	6 (11.3)	*χ*^2^ = 32.036 *P* < 0.001
	Cluster C	151 (37.2)	39 (22.2)	*χ*^2^ = 14.652 *P* < 0.001	109 (35.7)	28 (22.8)	42 (41.6)	11 (20.8)	*χ*^2^ = 13.866 *P* = 0.003

A higher prevalence of axis I disorders was observed in the suicide group compared with the control group (87.2% *v*. 61.9%, *χ*^2^ = 48.481, *P* < 0.001). Regarding specific diagnoses, depressive disorder was significantly more frequent in the suicide group (62.0% *v*. 40.3%, *χ*^2^ = 89.759, *P* < 0.001), as was psychotic disorder (10.6% *v*. 5.1%, *χ*^2^ = 14.474, *P* = 0.001). A higher prevalence of personality disorders was observed in the suicide group compared with the control group. Specifically, cluster A disorders were more frequent in the suicide group (32.3% *v*. 18.8%, *χ*^2^ = 11.486, *P* = 0.003), as were cluster B disorders (35.5% *v*. 12.5%, *χ*^2^ = 32.201, *P* < 0.001) and cluster C disorders (37.2% *v*. 22.2%, *χ*^2^ = 14.652, *P* = 0.001). These differences remained significant for both men and women. Additional results are presented in Table 1.

### Impulsivity and aggression by sex

**Fig. 2:**
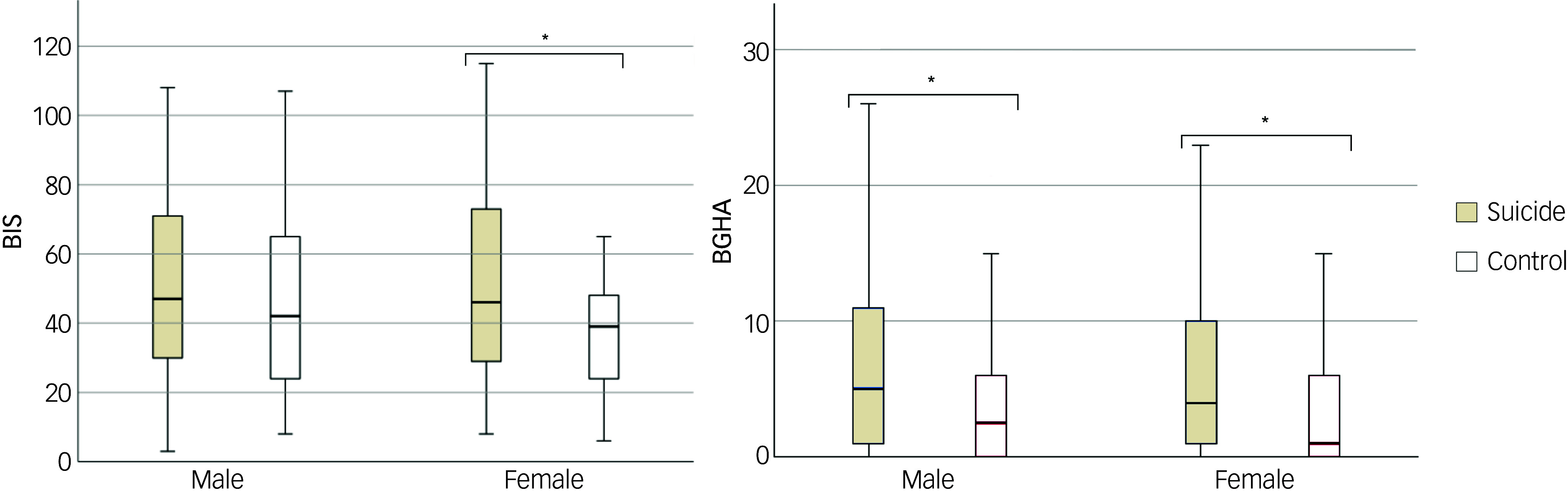
Boxplot of the distributions of Barratt Impulsiveness Scale (BIS) and Brown–Goodwin History of Aggression (BGHA) ratings by sex. *P < 0.05.

Mean impulsivity scores were higher in the suicide group compared with controls (51.3 *v*. 42.2, *P* = 0.002, *η*^2^ = 0.017). Similarly, BGHA ratings were elevated in the suicide group relative to controls (7.08 *v*. 4.14, *P* < 0.001, *η*^2^ = 0.028) (Fig. 2).

### Impulsivity and aggression by age

**Fig. 3:**
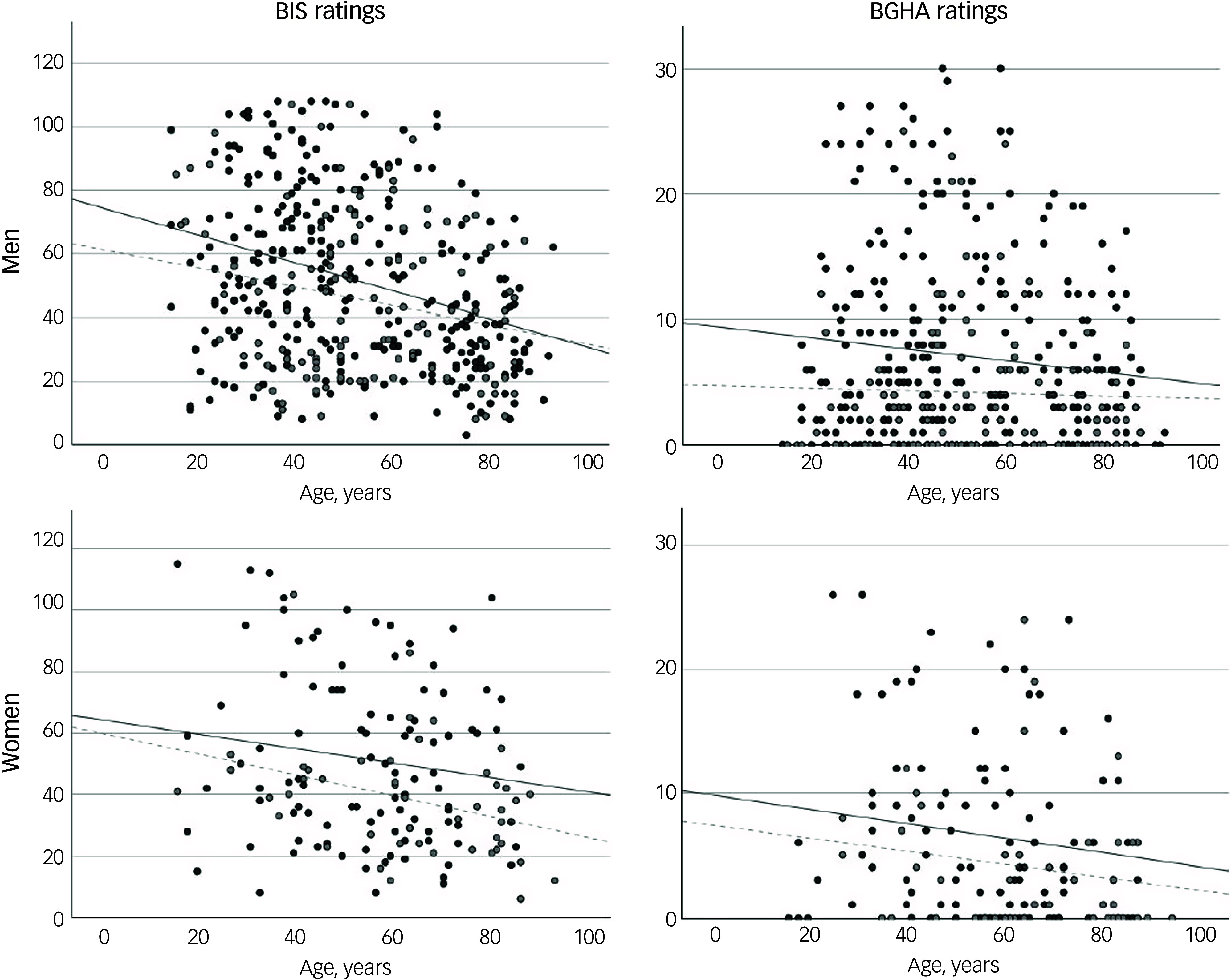
Correlations of age with Barratt Impulsiveness Scale (BIS) and Brown–Goodwin History of Aggression (BGHA) ratings by sex. Dark grey circles and continuous lines indicates individuals who died by suicide; light grey circles and dashed lines indicate controls.

In the analysis of variance, younger age was associated with higher BIS ratings (*P* < 0.001) with a medium effect size (*η*^2^ = 0.057), and with higher BGHA ratings (*P* = 0.001) with a low effect size (*η*^2^ = 0.023). There was no age × group interaction effect for BIS ratings (*P* = 0.176) or BGHA ratings (*P* = 0.798). BIS ratings decreased with age across all groups, showing significant correlations in the suicide male (*ρ* = −0.327, *P* < 0.001), control male (*ρ* = −0.153, *P* = 0.049) and control female (*ρ* = −0.375, *P* < 0.005) groups but not in the suicide female (*ρ* = −0.114, *P* = 0.127) group. BGHA ratings also declined with age in both suicide groups (suicide male: *ρ* = −0.129, *P* = 0.012; suicide female: *ρ* = −0.175, *P* = 0.038), but this trend was not observed in controls (control male: *ρ* = −0.012, *P* = 0.449; control female: *ρ* = −0.166, *p* = 0.138) (Fig. 3).

According to Fisher’s correction, age showed a significantly stronger correlation with BIS ratings than with BGHA ratings in the suicide male group (*P* = 0.009). No other significant differences between correlations were found. *Post hoc* comparisons using Bonferroni correction for age, BIS (impulsivity) and BGHA (aggression) variables are presented in Supplementary Table 2.

### Predictive models of death by suicide by sex

Variables were entered into the regression analysis (dependent variable: death by suicide) using a stepwise forward method. Although the explained variance in both models was modest (Nagelkerke’s *r*^2^ = 0.039 for men and *r*^2^ = 0.176 for women), the analysis revealed significant structural differences in suicide prediction between sexes. In men, only BGHA ratings emerged as a predictor of death by suicide (odds ratio 1.072, 95% CI: 1.032–1.112, *P* < 0.001). For women, predictors included younger age (odds ratio 0.968, 95% CI: 0.947–0.989, *P* < 0.05) and BIS ratings (odds ratio 1.020, 95% CI: 1.002–1.037, *P* < 0.05), with living with children serving as a protective factor (odds ratio 0.448, 95% CI: 0.208–0.966, *P* < 0.05).

Model fit was confirmed by Hosmer–Lemeshow test *P*-values (0.466 for men, 0.666 for women). AUC values indicated moderate predictive ability, with 0.615 for men and 0.692 for women in the final model (Table 2). Positive and negative predictive values for individual variables can be found in the Supplementary Material. Variance inflation factor values for all variables were below 5, indicating no collinearity among factors.

**Table 2:** Logistic regressions by sex through stepwise forward method with suicide as the dependent variable

	Odds ratio (95% CI)			
	Male	Female		
Variables	Step 1	Step 1	Step 2	Step 3
BGHA	1.072 (1.032–1.112)**			
Age		0.968 (0.947–0.989)*	0.973 (0.951–0.995)*	0.970 (0.948–0.993)*
BIS			1.020 (1.002–1.037)*	1.018 (1.001–1.036)*
Living with children				0.448 (0.208–0.966)*
Nagelkerke’s *R*^2^	0.039	0.090	0.139	0.176
Hosmer–Lemeshow test	0.466	0.217	0.549	0.666
AUC	0.615			0.692

AUC, area under the receiver operating characteristic curve; BGHA, Brown–Goodwin History of Aggression; BIS, Barratt Impulsivity Scale.

*P < 0.05; **P < 0.001.

## Discussion

We found that BIS and BGHA ratings were elevated in suicide cases across both sexes compared with controls, supporting hypothesis 1. Contrary to expectations, we found no sex-based differences in BIS and BGHA ratings within the overall sample or among suicide cases; thus, hypothesis 2 was rejected. Age was associated with lower BIS ratings across all groups, with significant reductions in the suicide male, control male and control female groups but not in the suicide female group. BGHA ratings decreased with age in both male and female suicide groups, with no significant correlation in the control groups, partially confirming hypothesis 3. Finally, the regression analysis showed impulsivity to be a stronger predictor of suicide in women, whereas aggression served as a more reliable predictor in men, supporting hypothesis 4. It is important to acknowledge that these findings were based on retrospective assessments of behaviours described as impulsive and aggressive by informants, rather than direct or objective measurements of these traits.

### Sex-based differences in impulsivity and aggression

Sex differences in impulsive and aggressive behaviours are well-documented. Studies have reported higher aggression levels in males, with aggression associated with the lethality of suicide attempts.^33^ Regarding impulsivity, Cross et al^34^ found in a meta-analysis that males scored higher on sensation-seeking (*d* = 0.41) and behavioural risk-taking tasks (*d* = 0.36) than females. Higher levels of dysfunctional impulsivity – characterised by rapid, inaccurate actions in situations where impulsivity is disadvantageous – and lower levels of functional impulsivity have been observed in individuals who die by suicide, particularly among males.^35^

Despite strong evidence linking impulsive and aggressive traits with suicide, particularly in men, our sample did not show elevated levels of these traits among male participants. Indeed, previous suicide studies have found that although lower impulsivity is generally reported for female compared with male cases, the proportion of individuals with ‘high impulsivity’ is similar across sexes and is often associated with substance misuse.^21^

These findings suggest that the relationships among impulsive–aggressive behaviours, sex and suicidal behaviour are complex and may be influenced by additional factors. For instance, one study on suicide attempts found that aggression and hostility distinguished women with mood disorders who made repeated attempts from those with only a single attempt, whereas in men, aggression and hostility did not vary with attempt frequency.^36^ Depressive disorders may also alter the link between impulsive–aggressive traits and fatal suicidal behaviour across sexes. Dalca et al^21^ found that among depressed male individuals who died by suicide, there was a higher prevalence of cluster B personality disorders and a greater proportion of highly impulsive individuals compared with controls. By contrast, depressed female individuals who died by suicide exhibited a phenotype labelled ‘non-impulsive aggression’, marked by aggression, cluster B personality disorders and lower impulsivity levels.

### Age-based differences in impulsivity and aggression

Our findings are consistent with those of McGirr et al,^23^ who observed that impulsive and aggressive behaviours in individuals who died by suicide decreased with age, whereas hostility remained stable. Using the Temperament and Character Inventory, McGirr et al also found that older individuals who died by suicide had higher scores for harm avoidance and persistence. These results are further supported by previous studies indicating that impulsivity is more pronounced in younger suicide groups.^16,37^

The interaction between young age and impulsivity has been associated with traits including heightened aggression and hostility,^38^ as well as a greater likelihood of using violent methods.^22^ A study on elderly groups found that individuals who died by suicide displayed higher levels of aggression compared with controls, although this difference diminished after the age of 65 years.^39^ In terms of psychopathology, impulsive and aggressive behaviours in young individuals who died by suicide have been linked to depression, cluster B personality disorders, and alcohol and drug misuse or dependence.^38^ This profile of young suicide victims highlights challenges in suicide prevention for this demographic, as risk and protective factors may vary with age. For instance, a study of depressed male individuals who died by suicide found higher levels of impulsivity and aggression specifically among younger individuals (ages 18–40 years).^40^

### Impulsive–aggression dimension: sex differences

Models explaining suicidal behaviour are used to consider the interplay of multiple factors, as it is recognised that individuals at risk of suicide may have certain predispositions that interact with triggering events.^41^ Impulsive–aggressive behaviour has been identified as both a predisposing and precipitating factor and is among the traits most strongly linked to suicidal behaviour.^5^ The associations of impulsivity, aggression and hostility in suicide are often captured in the term ‘impulsive–aggressive’, defined as a reaction that arises in response to deprivation or punishment and is accompanied by feelings such as frustration, irritability, fear or anger.^41^ Although impulsivity, aggression and hostility are interrelated predictors of suicidal behaviour, aggression and impulsivity do not always co-occur and represent distinct behaviours. For instance, aggression in suicide may be premeditated rather than impulsive.^42^

In our data, the interaction between impulsive and aggressive traits was not a reliable predictor of fatal suicidal behaviour. Instead, impulsivity and aggression individually proved to be better predictors of death by suicide in the stepwise regression analysis: BIS ratings in women and BGHA ratings in men. It is important to note, however, that the variance explained by these models was low, and the observed sex differences in BIS and BGHA ratings reflected general trends rather than a definitive pattern that distinctly differentiated men and women in terms of fatal suicidal behaviour.

The role of having children as a protective factor for women, but not for men, was notable. This finding was consistent with decades of research documenting this phenomenon,^43^ and the persistence of this trend underscores the influence of gender roles on suicidal behaviour and its underlying mechanisms. For instance, a psychological autopsy study found that in highly impulsive populations, social support did not serve as a protective factor against fatal suicidal behaviour.^44^ Owing to traditional gender roles, women often have closer support and caregiving networks than men. This may help to explain why impulsivity is a stronger predictor of death by suicide in women, as this trait may operate independently of the protective effects associated with caregiving roles.

Evidence suggests that individuals exposed to early adversity are at higher risk of developing pathological personality traits, emotional dysregulation, altered brain structures and executive function deficits, all of which are associated with suicidal behaviour.^5^ Together, these findings imply that the relationship between impulsive and aggressive traits and fatal suicidal behaviour may be indirect, with these traits acting as facilitators for other critical factors in suicide risk.

### Implications for suicide prevention

Results from this study highlight the relationship between impulsive and aggressive traits and suicidal behaviour, particularly in young adults, with these traits becoming less influential with age. Although the effect sizes in our study were modest, suicide prevention efforts could still benefit from considering the role of impulsive and aggressive features, especially in younger individuals. For instance, a recent Cochrane review of dialectical behaviour therapy, known for its effectiveness in reducing suicide attempts, found that it also reduced impulsivity.^45^ In addition, a clinical trial evaluating an intervention based on acceptance and commitment therapy for depressed individuals at high suicide risk reported significant reductions in anger expression.^46^ Although more research is needed to establish the extent to which these interventions directly mitigate suicide risk, incorporating evidence-based treatments that target impulsivity and aggression may be a valuable component of prevention strategies for high-risk populations. Overall, our findings underscore the importance of considering age- and sex-specific factors when assessing and intervening in suicide risk.

### Limitations

Limitations of this study arise from the psychological autopsy methodology, according to which information was collected retrospectively from informants. Although many prior studies have validated this methodology, including its ability to assess behavioural aspects such as impulsive and aggressive traits, it is important to acknowledge that the ratings reflect informant perceptions rather than direct or objective measures of behaviour. In particular, informants for individuals who died by suicide may have been more likely to retrospectively emphasise impulsive or aggressive behaviours, potentially introducing recall or perception bias. Another limitation was that interrater reliability was not formally assessed for informant-based measures of impulsivity and aggression. However, in psychological autopsy studies, these evaluations are inherently subjective, as they rely on retrospective reports from informants rather than direct clinical assessments. To improve reliability, consensus meetings among trained psychiatrists and psychologists were conducted to resolve discrepancies in the diagnostic process. We also established a control group consisting of individuals with natural and accidental causes of death, as it has been suggested that the characteristics of individuals with non-suicidal causes of death may differ from those of people who die by suicide.^9^ In addition, our study was subject to selection bias, as participation was limited to individuals with living relatives who were willing to take part in the study, excluding cases in which relatives declined participation or the deceased individual had no surviving family members. Furthermore, there was a substantial loss of informants from the total number of suicide cases initially considered, which may have introduced additional selection bias. Finally, the limited number of variables included in the analyses may have restricted the scope of the findings and their generalisability.

## Supporting information

Sanz-Gómez et al. supplementary material 1 Sanz-Gómez et al. supplementary material

Sanz-Gómez et al. supplementary material 2 Sanz-Gómez et al. supplementary material

Sanz-Gómez et al. supplementary material 3 Sanz-Gómez et al. supplementary material

## Data Availability

The data that support the findings of this study are available on request from the corresponding author, D.d.-l.-V.-S. The data are not accessible to the general public owing to their inclusion of sensitive information, the protection of which is enshrined in Spanish legislation.

## References

[1] World Health Organization (WHO). Suicide in the World: Global Health Estimates. WHO, 2019 (https://apps.who.int/iris/bitstream/handle/10665/326948/WHO-MSD-MER-19.3-eng.pdf).

[2] Sutar R , Kumar A , Yadav V. Suicide and prevalence of mental disorders: a systematic review and meta-analysis of world data on case-control psychological autopsy studies. Psychiatry Res 2023; 329: 115492. 37783094 10.1016/j.psychres.2023.115492

[3] Cho SEJ , Na KS , Cho SEJ , Im JS , Kang SG. Geographical and temporal variations in the prevalence of mental disorders in suicide: systematic review and meta-analysis. J Affect Disord 2016; 190: 704–13. 26600412 10.1016/j.jad.2015.11.008

[4] Favril L , Yu R , Uyar A , Sharpe M , Fazel S. Risk factors for suicide in adults: systematic review and meta-analysis of psychological autopsy studies. Evid Based Ment Health 2022; 25: 148–55. 36162975 10.1136/ebmental-2022-300549 PMC9685708

[5] Turecki G , Brent DA , Gunnell D , O’Connor RC , Oquendo MA , Pirkis J , et al. Suicide and suicide risk. Nat Rev Dis Prim 2019; 5: 74. 31649257 10.1038/s41572-019-0121-0

[6] National Center for Health Statistics. Health, United States, 2020-2021: Suicide. National Center for Health Statistics, 2023 (https://www.cdc.gov/nchs/hus/topics/suicide.htm). 36888733

[7] Arsenault-Lapierre G , Kim CD , Turecki G. Psychiatric diagnoses in 3275 suicides: a meta-analysis. BMC Psychiatry 2004; 4: 37. 15527502 10.1186/1471-244X-4-37 PMC534107

[8] Steele IH , Thrower N , Noroian P , Saleh FM. Understanding suicide across the lifespan: a United States perspective of suicide risk factors, assessment and management. J Forensic Sci 2018; 63: 162–71. 28639299 10.1111/1556-4029.13519

[9] De Leo D , Draper BM , Snowdon J , Kõlves K. Suicides in older adults: a case-control psychological autopsy study in Australia. J Psychiatr Res 2013; 47: 980–8. 23522934 10.1016/j.jpsychires.2013.02.009

[10] Giner L , Blasco-Fontecilla H , Mercedes Perez-Rodriguez M , Garcia-Nieto R , Giner J , Guija JA , et al. Personality disorders and health problems distinguish suicide attempters from completers in a direct comparison. J Affect Disord 2013; 151: 474–83. 23859005 10.1016/j.jad.2013.06.029

[11] Van Orden K , Conwell Y. Suicides in late life. Curr Psychiatry Rep 2011; 13: 234–41. 21369952 10.1007/s11920-011-0193-3 PMC3085020

[12] Brezo J , Paris J , Turecki G. Personality traits as correlates of suicidal ideation, suicide attempts, and suicide completions: a systematic review. Acta Psychiatr Scand 2006; 113: 180–206. 16466403 10.1111/j.1600-0447.2005.00702.x

[13] Stanford MS , Mathias CW , Dougherty DM , Lake SL , Anderson NE , Patton JH. Fifty years of the Barratt Impulsiveness Scale: an update and review. Pers Individ Differ 2009; 47: 385–95.

[14] Gvion Y , Apter A. Aggression, impulsivity, and suicide behavior: a review of the literature. Arch Suicide Res 2011; 15: 93–112. 21541857 10.1080/13811118.2011.565265

[15] Chamorro J , Bernardi S , Potenza MN , Grant JE , Marsh R , Wang S , et al. Impulsivity in the general population: a national study. J Psychiatr Res 2012; 46: 994–1001. 22626529 10.1016/j.jpsychires.2012.04.023 PMC3564492

[16] Kim CD , Lesage A , Seguin M , Chawky N , Vanier C , Lipp O , et al. Patterns of co-morbidity in male suicide completers. Psychol Med 2003; 33: 1299–309. 14580083 10.1017/s0033291703008146

[17] McGirr A , Alda M , Séguin M , Cabot S , Lesage A , Turecki G. Familial aggregation of suicide explained by cluster B traits: a three-group family study of suicide controlling for major depressive disorder. Am J Psychiatry 2009; 166: 1124–34. 19755577 10.1176/appi.ajp.2009.08111744

[18] Turecki G. Dissecting the suicide phenotype: the role of impulsive-aggressive behaviours. J Psychiatry Neurosci 2005; 30: 398–408. 16327873 PMC1277022

[19] Parra-Uribe I , Blasco-Fontecilla H , Garcia-Parés G , Martínez-Naval L , Valero-Coppin O , Cebrià-Meca A , et al. Risk of re-attempts and suicide death after a suicide attempt: a survival analysis. BMC Psychiatry 2017; 17: 163. 28472923 10.1186/s12888-017-1317-z PMC5415954

[20] Irigoyen-Otiñano M , Puigdevall-Ruestes M , Mur-Laín M , González-Pinto A , Portella MJ , Baca-García E , et al. Absence of association between the level of lethality and the recidivism of suicide attempts in a Spanish province. Actas Esp Psiquiatr 2019; 47: 179–89. 31648340

[21] Dalca IM , McGirr A , Renaud J , Turecki G. Gender-specific suicide risk factors: a case-control study of individuals with major depressive disorder. J Clin Psychiatry 2013; 74: 1209–15. 24434089 10.4088/JCP.12m08180

[22] Dumais A , Lesage AD , Lalovic A , Séguin M , Tousignant M , Chawky N , et al. Is violent method of suicide a behavioral marker of lifetime aggression? Am J Psychiatry 2005; 162: 1375–8. 15994723 10.1176/appi.ajp.162.7.1375

[23] McGirr A , Renaud J , Bureau A , Seguin M , Lesage A , Turecki G. Impulsive-aggressive behaviours and completed suicide across the life cycle: a predisposition for younger age of suicide. Psychol Med 2008; 38: 407–17. 17803833 10.1017/S0033291707001419

[24] Instituto Nacional de Estadística (INE). Sevilla: Población por municipios y sexo [Seville: Population by Municipality and Sex]. INE, 2022.

[25] Conner KR , Chapman BP , Beautrais AL , Brent DA , Bridge JA , Conwell Y , et al. Introducing the psychological autopsy methodology checklist. Suicide Life Threat Behav 2021; 51: 673–83. 33559215 10.1111/sltb.12738 PMC8378509

[26] First M , Spitzer RL , Gibbon M , Williams JBW. Entrevista clínica estructurada para los trastornos del eje I del DSM-IV: SCID-I. Versión Clínica Ed Masson Barcelona [Structured clinical interview for DSM-IV Axis I disorders. Clinical Version. Ed Masson Barcelona]. Vol. 2. Elsevier Masson, 1999.

[27] First MB , Spitzer RL , Gibbon M , Williams JBW. The structured clinical interview for DSM-III-R personality disorders (SCID-II). Part II: multi-site test-retest reliability study. J Pers Disord 1995; 9: 83–91.

[28] Schneider B , Maurer K , Sargk D , Heiskel H , Weber B , Frölich L , et al. Concordance of DSM-IV Axis I and II diagnoses by personal and informant’s interview. Psychiatry Res 2004; 127: 121–36. 15261711 10.1016/j.psychres.2004.02.015

[29] Oquendo MA , Baca-Garcia E , Graver R , Morales M , Montalvan V , Mann JJ. Spanish adaptation of the Barratt impulsiveness scale (BIS-11). Eur J Psychiatry 2001; 15: 147–55.

[30] Brown GL , Goodwin FK. Cerebrospinal fluid correlates of suicide attempts and aggression. Ann N Y Acad Sci 1986; 487: 175–88. 2436532 10.1111/j.1749-6632.1986.tb27897.x

[31] Oquendo MA , Currier D , Mann JJ. Prospective studies of suicidal behavior in major depressive and bipolar disorders: what is the evidence for predictive risk factors? Acta Psychiatr Scand 2006; 114: 151–8. 16889585 10.1111/j.1600-0447.2006.00829.x

[32] Sanz-Gómez S , Alacreu-Crespo A , Guija JA , Giner-Jiménez L. Reliability and validity of proxy reports of impulsivity and aggression: an evidence-based assessment approach to psychological autopsy methods. Span J Psychiatry Ment Health 2025; 18: 28–33. 37979784 10.1016/j.sjpmh.2023.10.003

[33] Carballo JJ , García-Nieto R , Harkavy-Friedman J , de Leon-Martinez V , Baca-García E. Aggressiveness across development and suicidal behavior in depressed patients. Arch Suicide Res 2014; 18: 39–49. 24579919 10.1080/13811118.2013.801808

[34] Cross CP , Copping LT , Campbell A. Sex differences in impulsivity: a meta-analysis. Psychol Bull 1996; 119: 410–21. 21219058 10.1037/a0021591

[35] Zhang J , Lin L. The moderating effects of impulsivity on Chinese rural young suicide. J Clin Psychol 2014; 70: 579–88. 24002993 10.1002/jclp.22039

[36] Papadopoulou A , Efstathiou V , Christodoulou C , Gournellis R , Papageorgiou C , Douzenis A , et al. Psychiatric diagnosis, gender, aggression, and mode of attempt in patients with single versus repeated suicide attempts. Psychiatry Res 2020; 284: 112747. 31927168 10.1016/j.psychres.2020.112747

[37] Brent DA , Apter A. Adolescent suicide and suicidal behavior: a time to assess and a time to treat. Isr J Psychiatry Relat Sci 2003; 40: 159–62. 14619673

[38] Zouk H , Tousignant M , Seguin M , Lesage A , Turecki G. Characterization of impulsivity in suicide completers: clinical, behavioral and psychosocial dimensions. J Affect Disord 2006; 92: 195–204. 16545465 10.1016/j.jad.2006.01.016

[39] Conner KR , Conwell Y , Duberstein PR , Eberly S. Aggression in suicide among adults age 50 and over. Am J Geriatr Psychiatry 2004; 12: 37–42. 14729557

[40] Dumais A , Lesage AD , Alda M , Rouleau G , Dumont M , Chawky N , et al. Risk factors for suicide completion in major depression: a case-control study of impulsive and aggressive behaviors in men. Am J Psychiatry 2005; 162: 2116–24. 16263852 10.1176/appi.ajp.162.11.2116

[41] Oquendo MA , Mann JJ. The biology of impulsivity and suicidality. Psychiatr Clin North Am 2000; 23: 11–25. 10729928 10.1016/s0193-953x(05)70140-4

[42] Keilp JG , Gorlyn M , Oquendo MA , Brodsky B , Ellis SP , Stanley B , et al. Aggressiveness, not impulsiveness or hostility, distinguishes suicide attempters with major depression. Psychol Med 2006; 36: 1779–88. 16959059 10.1017/S0033291706008725

[43] Canetto SS. Women and suicidal behavior: a cultural analysis. Am J Orthopsychiatry 2008; 78: 259–66. 18954189 10.1037/a0013973

[44] Zhang J , Lin L. The moderating effect of social support on the relationship between impulsivity and suicide in rural China. Community Ment Health J 2015; 51: 585–90. 25540027 10.1007/s10597-014-9811-y

[45] Storebø OJ , Stoffers-Winterling JM , Völlm BA , Kongerslev MT , Mattivi JT , Jørgensen MS , et al. Psychological therapies for people with borderline personality disorder. Cochrane Database Syst Rev 2020; 5: CD012955. 32368793 10.1002/14651858.CD012955.pub2 PMC7199382

[46] Ducasse D , Jaussent I , Arpon-Brand V , Vienot M , Laglaoui C , Béziat S , et al. Acceptance and commitment therapy for the management of suicidal patients: a randomized controlled trial. Psychother Psychosom 2018; 87: 211–22. 29874680 10.1159/000488715

