# Supplementary material for: Impulsivity and aggression in suicide across age and sex: case–control study
Source: BJPsych Open. 2025 Aug 1;11(5):e167. doi: 10.1192/bjo.2025.10076 (PMC12344428; doi:10.1192/bjo.2025.10076)
Supplement: Sanz-Gómez et al. supplementary material 1 — Sanz-Gómez et al. supplementary material [file S2056472425100768sup001.docx]

Supplementary material 3. Individual positive and negative predictive values of variables introduced in the regression models

|  | Variable | Cut-off point | AUC (95% CI) | PPV | NPV |
| --- | --- | --- | --- | --- | --- |
| Males | BIS | 28 | .575 (.515-.635) | 0.79 | 0.32 |
|  | BGHA | 35 | .619 (.563-.675) | 0.57 | 0.63 |
|  | Age | 44 | .477 (.419-.535) | 0.64 | 0.24 |
|  | Living with children | 1 | .495 (.435-555) | 0.01 | 0.99 |
| Females | BIS | 56 | .638 (.550-.725) | 0.43 | 0.89 |
|  | BGHA | 15 | .633 (.541-724) | 0.72 | 0.51 |
|  | Age | 51 | .388 (.294-.481) | 0.61 | 0.26 |
|  | Living with children | 1 | .510 (.416-.604) | 0.02 | 1 |
