# Supplementary material for: Impulsivity and aggression in suicide across age and sex: case–control study
Source: BJPsych Open. 2025 Aug 1;11(5):e167. doi: 10.1192/bjo.2025.10076 (PMC12344428; doi:10.1192/bjo.2025.10076)
Supplement: Sanz-Gómez et al. supplementary material 2 — Sanz-Gómez et al. supplementary material [file S2056472425100768sup002.docx]

Supplementary Table 1. Recruitment process and participation breakdown by year

| **Year** | **Total suicides in the area*** | **Number of families agreed to participate** | **Percentage over total suicides** |
| --- | --- | --- | --- |
| 2006 | 141 | 77 | 54.6% |
| 2007 | 116 | 87 | 75.0% |
| 2008 | 163 | 104 | 63.8% |
| 2009 | 125 | 29 | 23.2% |
| 2010 | 145 | 35 | 24.1% |
| 2011 | 121 | 34 | 28.1% |
| 2012 | 156 | 22 | 14.1% |
| 2013 | 161 | 24 | 14.9% |
| Total | 1128 | 412 | 36.5% |

* Suicides registered in the province of Seville. Data provided by the INE.
