# Supplementary material for: Impulsivity and aggression in suicide across age and sex: case–control study
Source: BJPsych Open. 2025 Aug 1;11(5):e167. doi: 10.1192/bjo.2025.10076 (PMC12344428; doi:10.1192/bjo.2025.10076)
Supplement: Sanz-Gómez et al. supplementary material 3 — Sanz-Gómez et al. supplementary material [file S2056472425100768sup003.docx]

| Appendix. Multiple Comparisons (Bonferroni Test) | | | | | | | |
| --- | --- | --- | --- | --- | --- | --- | --- |
|  | (I) Group | (J) Group | Mean Difference (I-J) | SE | Sig. | 95% CI | |
|  |  |  |  |  |  | Lower bound | Upper bound |
| Age | Suicide Male | Control Male | -1,78971 | 1,98985 | 1,000 | -7,0576 | 3,4782 |
|  |  | Suicide Female | -2,65126 | 2,13872 | 1,000 | -8,3132 | 3,0107 |
|  |  | Control Female | -9,88160^*^ | 2,77239 | ,002 | -17,2211 | -2,5420 |
|  | Control Male | Suicide Male | 1,78971 | 1,98985 | 1,000 | -3,4782 | 7,0576 |
|  |  | Suicide Female | -,86155 | 2,50156 | 1,000 | -7,4841 | 5,7610 |
|  |  | Control Female | -8,09189 | 3,06102 | ,051 | -16,1955 | ,0118 |
|  | Suicide Female | Suicide Male | 2,65126 | 2,13872 | 1,000 | -3,0107 | 8,3132 |
|  |  | Control Male | ,86155 | 2,50156 | 1,000 | -5,7610 | 7,4841 |
|  |  | Control Female | -7,23034 | 3,15982 | ,135 | -15,5956 | 1,1349 |
|  | Control Female | Suicide Male | 9,88160^*^ | 2,77239 | ,002 | 2,5420 | 17,2211 |
|  |  | Control Male | 8,09189 | 3,06102 | ,051 | -,0118 | 16,1955 |
|  |  | Suicide Female | 7,23034 | 3,15982 | ,135 | -1,1349 | 15,5956 |
| BIS (impulsivity) | Suicide Male | Control Male | 7,02340 | 2,77837 | ,070 | -,3325 | 14,3794 |
|  |  | Suicide Female | ,37037 | 2,98086 | 1,000 | -7,5217 | 8,2624 |
|  |  | Control Female | 14,07592^*^ | 3,83008 | ,002 | 3,9355 | 24,2164 |
|  | Control Male | Suicide Male | -7,02340 | 2,77837 | ,070 | -14,3794 | ,3325 |
|  |  | Suicide Female | -6,65303 | 3,48742 | ,342 | -15,8863 | 2,5802 |
|  |  | Control Female | 7,05252 | 4,23628 | ,579 | -4,1634 | 18,2684 |
|  | Suicide Female | Suicide Male | -,37037 | 2,98086 | 1,000 | -8,2624 | 7,5217 |
|  |  | Control Male | 6,65303 | 3,48742 | ,342 | -2,5802 | 15,8863 |
|  |  | Control Female | 13,70555^*^ | 4,37176 | ,011 | 2,1310 | 25,2801 |
|  | Control Female | Suicide Male | -14,07592^*^ | 3,83008 | ,002 | -24,2164 | -3,9355 |
|  |  | Control Male | -7,05252 | 4,23628 | ,579 | -18,2684 | 4,1634 |
|  |  | Suicide Female | -13,70555^*^ | 4,37176 | ,011 | -25,2801 | -2,1310 |
| BGHA (Aggression) | Suicide Male | Control Male | 2,97593^*^ | ,73238 | ,000 | 1,0370 | 4,9148 |
|  |  | Suicide Female | ,37897 | ,78712 | 1,000 | -1,7048 | 2,4628 |
|  |  | Control Female | 3,15218^*^ | 1,02017 | ,013 | ,4514 | 5,8530 |
|  | Control Male | Suicide Male | -2,97593^*^ | ,73238 | ,000 | -4,9148 | -1,0370 |
|  |  | Suicide Female | -2,59696^*^ | ,92029 | ,030 | -5,0333 | -,1606 |
|  |  | Control Female | ,17625 | 1,12611 | 1,000 | -2,8050 | 3,1575 |
|  | Suicide Female | Suicide Male | -,37897 | ,78712 | 1,000 | -2,4628 | 1,7048 |
|  |  | Control Male | 2,59696^*^ | ,92029 | ,030 | ,1606 | 5,0333 |
|  |  | Control Female | 2,77321 | 1,16245 | ,104 | -,3042 | 5,8507 |
|  | Control Female | Suicide Male | -3,15218^*^ | 1,02017 | ,013 | -5,8530 | -,4514 |
|  |  | Control Male | -,17625 | 1,12611 | 1,000 | -3,1575 | 2,8050 |
|  |  | Suicide Female | -2,77321 | 1,16245 | ,104 | -5,8507 | ,3042 |
